# The NISTmAb Reference Material 8671 value assignment, homogeneity, and stability

**DOI:** 10.1007/s00216-017-0800-1

**Published:** 2018-02-07

**Authors:** John E. Schiel, Abby Turner, Trina Mouchahoir, Katharina Yandrofski, Srivalli Telikepalli, Jason King, Paul DeRose, Dean Ripple, Karen Phinney

**Affiliations:** 1 National Institute of Standards and Technology, Institute for Bioscience and Biotechnology Research, 9600 Gudelsky Dr, Rockville, MD 20850 USA; 2 Present Address: 55 Watkins Mill Rd., Gaithersburg, MD USA; 3 National Institute of Standards and Technology, 100 Bureau Drive, Gaithersburg, MD 20899 USA

**Keywords:** Reference Material, NISTmAb, Monoclonal antibody, Biotherapeutic, Biopharmaceutical, System suitability, Biosimilar

## Abstract

The NISTmAb Reference Material (RM) 8671 is intended to be an industry standard monoclonal antibody for pre-competitive harmonization of best practices and designing next generation characterization technologies for identity, quality, and stability testing. It must therefore embody the quality and characteristics of a typical biopharmaceutical product and be available long-term in a stable format with consistent product quality attributes. A stratified sampling and analysis plan using a series of qualified analytical and biophysical methods is described that assures RM 8671 meets these criteria. Results for the first three lots of RM 8671 highlight the consistency of material attributes with respect to size, charge, and identity. RM 8671 was verified to be homogeneous both within and between vialing lots, demonstrating the robustness of the lifecycle management plan. It was analyzed in concert with the in-house primary sample 8670 (PS 8670) to provide a historical link to this seminal material. RM 8671 was verified to be fit for its intended purpose as a technology innovation tool, external system suitability control, and cross-industry harmonization platform.

**Electronic supplementary material:**

The online version of this article (10.1007/s00216-017-0800-1) contains supplementary material, which is available to authorized users.

**Graphical abstract:**
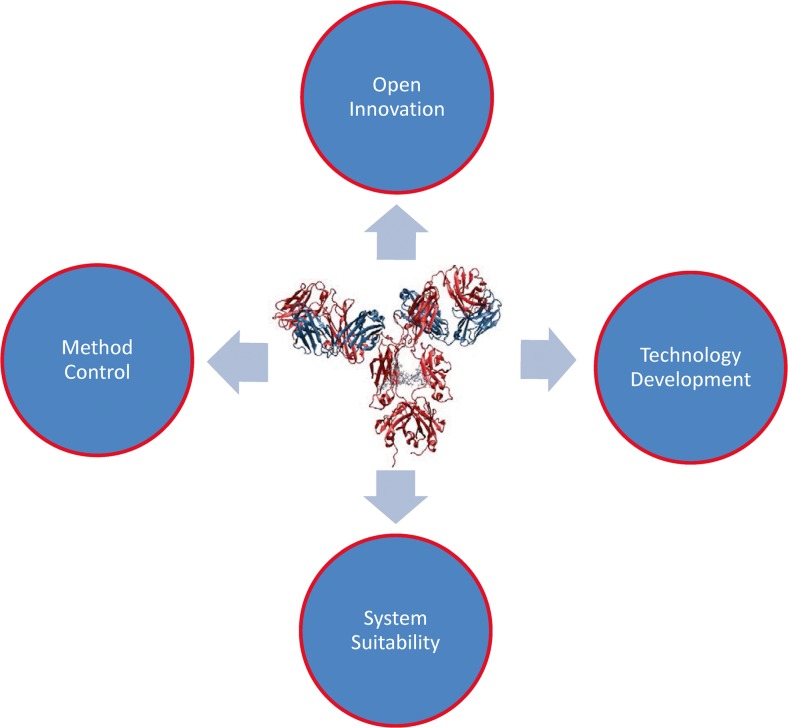
The NISTmAb Reference Material (RM) 8671 is intended to be an industry standard monoclonal antibody for pre-competitive harmonization of best practices and designing next generation characterization technologies for identity, quality, and stability testing.

## Introduction

The NISTmAb Reference Material (RM) 8671 is intended as a product-agnostic control to support biopharmaceutical innovation, and was therefore developed under a quality system of similar rigor to marketed biotechnology products. Quality of biotechnology products is defined as the suitability of the drug substance or drug product for its intended use [1]. This term includes product quality attributes such as quantity, identity, biological activity, and purity. Appropriate specification setting to ensure quality for biotechnology products must be underpinned by rigorous characterization of physicochemical, biophysical, and immunochemical properties [2]. The most immediate utility of the NISTmAb is for physicochemical/biophysical analysis technology development, method control, and harmonization of best practices. An expansive and continuously growing historical characterization dataset has been compiled as a foundation for these intended uses [3–8], a subset of which has been refined and qualified for quality monitoring [9–12], and used herein for quality attribute value assignment as part of the NISTmAb lifecycle management plan.

An RM must be established to be fit for its intended use in measurement or in examination of nominal properties, sufficiently homogeneous and stable with respect to specified properties, and available long-term with consistent product quality attributes to serve as a sustainable industry standard. Property values may be assigned to a given RM through a variety of measurement modes, the results of which influence the value designation (certified, reference, or informational as described in) [10, 13]. A series of method-specific protocols, each of which has been recognized by the community as common measurement methods for mAbs, were qualified to be suitable for NISTmAb measurement in the preceding papers of this series using the in-house NISTmAb primary sample (PS 8670) [9–12]. The physicochemical property values were then assigned to each individual lot of RM 8671 using the qualified methods and a stratified sampling and analysis plan, thereby allowing simultaneous evaluation of intra-lot homogeneity. The following discussion also shows inter-lot homogeneity with respect to each property value through analysis of three lots of RM 8671. The consistent performance of each lot provides confidence that the designated process and lifecycle management plan will produce consistent product quality attributes for current and future lots of RM 8671.

The NISTmAb RM 8671 is intended to be a widely available metric used to evaluate current capabilities and compare emerging technologies to define analytical and biophysical capabilities. Each individual lot of RM 8671, shown herein to demonstrate minimal variation in product quality attributes, is accompanied by a lot-specific Report of Investigation that describes the properties of the RM and its intended use. The current publication series is a thorough expansion on the RM 8671 Report of Investigation, and affords an opportunity to report more detailed procedures and results obtained from the value assignment exercise. This comprehensive report is a necessity to demonstrate RM quality, fully describe current state-of-the-art measurement capabilities, and serves as a means to continually evaluate best practices, promote innovative approaches, and inform regulatory paradigms as technology advances.

## Materials and methods

### NISTmAb PS 8670 and RM 8671

Vialing of NISTmAb primary sample (PS) 8670 and RM 8671 (lots 14HB-D-001, 14HB-D-002, and 14HB-D-003) are described in the first publication of this series [10]. Samples from each RM 8671 lot for homogeneity and value assignment, thaw/freeze (T/F) stability, and accelerated stability analysis were reserved as indicated in the Electronic Supplementary Material (ESM).

### UV-vis optical absorbance analysis

To establish homogeneity and assign reference values, ten vials were analyzed from each of lot 14HB-D-001, 14HB-D-002, and 14HB-D-003 for a total of thirty vials (no intra-vial replicates). Thaw/freeze stability with respect to concentration was assessed using vials from lot 14HB-D-001 that had undergone 1 or 5 thaw/freeze cycles at either −80 °C or −20 °C, for a total of four (4) vials. The zero time point and three vials from the 1, 7, and 28 day time points were evaluated from each accelerated stability temperature (4 °C, room temperature, and 40 °C).

Reference value assignment was performed using a nominal 0.5 mm path length cuvette from Starna Cells, Inc. (Atascadero, CA) and constructed from Spectrosil fused silica. Primary decadic attenuance measurements, which is analogous to absorbance except without light scatter correction, of the mAb were measured using the NIST Material Measurement Laboratory (MML) Transfer Spectrophotometer (Cary 6000i spectrometer (Agilent)). Traceability is to the decadic logarithm of the derived International System of Units (SI unit) of regular spectral transmittance (expressed as decadic attenuance) via the national reference instrument for optical absorbance (HAS-2) [14, 15]. Traceability is achieved using transfer standards SRMs 2034 *Holmium Oxide Solution Wavelength Standard (240 nm to 650 nm)*; 930, 1930 and 2930 *Glass Filters for Spectrophotometry*; and 2031 *Metal-on Fused--Silica Neutral Density Filters (250 nm to 635 nm)*. Calibration of the wavelength scale was done using SRM 2034. The Cary instrument was allowed to warm up for 30 min before measurements were taken to stabilize the UV lamp. The instrument was operated in double beam mode with a decreased slit height, temperature of 22 °C, and a spectral bandwidth of 0.8 nm, which are the typical operation conditions used to match the HAS II reference spectrophotometer. The decadic attenuance spectra were measured from 240 nm to 340 nm, at a scan rate of 20 nm/min and wavelength interval of 0.5 nm.

Decadic attenuance measurements at 280 nm (D280) of the samples were made using the same cuvette as the blank (formulation buffer prepared as indicated in ESM). Note that the NISTmAb sample was measured directly without further dilution or other preparation steps. The Blank D280 was subtracted from each RM sample D280 to provide a Corrected D280 (*D*_corr_). Concentration of the NISTmAb was determined utilizing a theoretical extinction coefficient (*ε*) of 1.42 (mL mg^−1^ cm^−1^) [16]. The equation used for calculation of the concentration (*C*) was

1 $$ C=\frac{D_{corr}}{\varepsilon \bullet b} $$

with a path length (*b*) of 0.05092 cm.

### Physicochemical methods

Capillary zone electrophoresis (CZE), size exclusion chromatography (SEC), reducing sodium dodecyl sulfate capillary electrophoresis (rCE-SDS), and non-reducing sodium dodecyl sulfate capillary electrophoresis (nrCE-SDS) were performed according to the qualified methods as described in publications 2 and 3 of this series [11, 12]. To establish homogeneity and assign reference values, one 150 μL vial fraction (prepared as described in the ESM) was analyzed from three racks (racks 1, 50, 90) of each lot of RM 8671 (*n* = 3 vials per lot). The standard protocol for each lot was to perform triplicate repeatability on one vial and individual measurements on two additional vials for physicochemical value assignment. In some cases, deviations from this sampling sequence were required to allow analysis of all three lots within 24 h as described in ESM. Thaw/Freeze stability samples were analyzed from lot 14HB-D-001 vials that had undergone 1 or 5 thaw/freeze cycles at either −80 °C or −20 °C, for a total of 4 samples. Accelerated stability samples were analyzed from lot 14HB-D-001 vials that had undergone incubation at 4 °C, room temperature, or 40 °C for zero, 7, or 28 days for a total of 7 samples; an additional time point of 1 day at each temperature was analyzed by SEC. Method performance was evaluated on the day of analysis by injections of the method-specific instrument quality control standard (IQ) and PS 8670 as the system suitability standard that bracketed injections of RM 8671. The generic injection sequence was: Blank - IQ - PS 8670 - test samples (no more than 10) - IQ - PS 8670 – Blank, and was repeated as necessary to analyze all samples. Instrument qualification and system suitability controls were required to pass method performance criteria as outlined during method qualification [11, 12]. Data analysis for each method was performed as described in the requisite qualification paper [9, 11, 12]. Combined standard uncertainty (*u*_*c*_) for RM 8671 samples were calculated as described in ESM. The combined standard uncertainty is intended to represent, at the level of one standard deviation, the effect of the combined components of uncertainty including Type A measurement uncertainty and Type B components related to intermediate precision as observed during method qualification.

### Informational value methods

Flow imaging (FI) and dynamic light scattering (DLS) were performed according to the optimized methods described in publication 3 of this series [12]. To establish homogeneity and assign informational values, six vials (two obtained from each rack numbered 1, 50, and 90) from each of lot 14HB-D-001, 14HB-D-002 and 14HB-D-003 were analyzed by FI. Three vials from each lot 14HB-D-001, 14HB-D-002 and 14HB-D-003 were used for homogeneity assessment by DLS. A total of twelve vials were evaluated from the 14HB-D-001 lot that had undergone 1 or 5 thaw/freeze cycles at either −80 °C or at −20 °C. The same number of vials containing PS 8670, stressed in an identical fashion, was also used for comparison. A total of 22 vials from the 14HB-D-001 lot were used for a stability study. This sample set contained the following: four zero time point vials, six vials stored at 4 °C for 7 days or 28 days, six vials stored at room temperature for 7 days or 28 days, and six vials stored at 40 °C for 7 days or 28 days. The formulation buffer (described in ESM) alone was also stressed under the same conditions and assessed for subvisible particle concentration. Method performance was evaluated on the day of analysis by injection of the method-specific instrument quality control standard (IQ) and PS 8670 as the system suitability standard. Data collection and analysis for each method was performed as described previously [12].

### Identity

Peptide mapping using reversed phase ultrahigh performance liquid chromatography coupled to UV-Visible and tandem mass spectrometry detection (LC-UV-MS/MS) was used to confirm the primary amino acid sequence. LC-UV-MS/MS was performed according to the method as described in publication 4 of this series [9]. To confirm identity, one 150 μL vial fraction (prepared as described in ESM) was analyzed from rack 1 of each lot of RM 8671 (14HB-D-001, 14HB-D-002, 14HB-D-003). Method performance was evaluated on the day of analysis by parallel sample preparation and analysis of PS 8670. Data collection and analysis for each method was performed as described previously [9].

## Results

### Reference values

#### UV-vis optical absorbance analysis

Concentration of protein material is often determined using UV-Visible spectrophotometry wherein the measured absorbance is assumed to be equivalent to the decadic attenuance. The decadic attenuance, D, is computed as the negative logarithm (base 10) of the transmittance, and is analogous to absorbance except for the inclusion of scattering and luminescence effects upon the radiant power exiting the sample [17]. Concentrations reported herein are based on decadic attenuance at 280 nm (D280) with no scatter or luminescence correction. Although the use of decadic attenuance results in an “apparent concentration,” it is most reflective of the experiments commonly performed by the end user (e.g. no scatter or luminescence correction). It should be noted, however, that the D320 has been reported as a means to correct for scattering at 280 nm [18]. Proteins absorb little to no light at 320 nm, and therefore the D320 represents scattered rather than absorbed light. Correction at 280 nm is performed via the Rayleigh scattering equation (intensity of scattered light is proportional to λ^−4^). This correction becomes increasingly important under formulation conditions inducing colloidal instability. In the current study, D320 was determined to be 0.00222, 0.00287, and 0.00184 (14HB-D-001, 14HB-D-002, 14HB-D-003, respectively) absorbance units which represents a small contribution and is in agreement with the low level of particulates observed using flow imaging as discussed below.

**Fig. 1:**
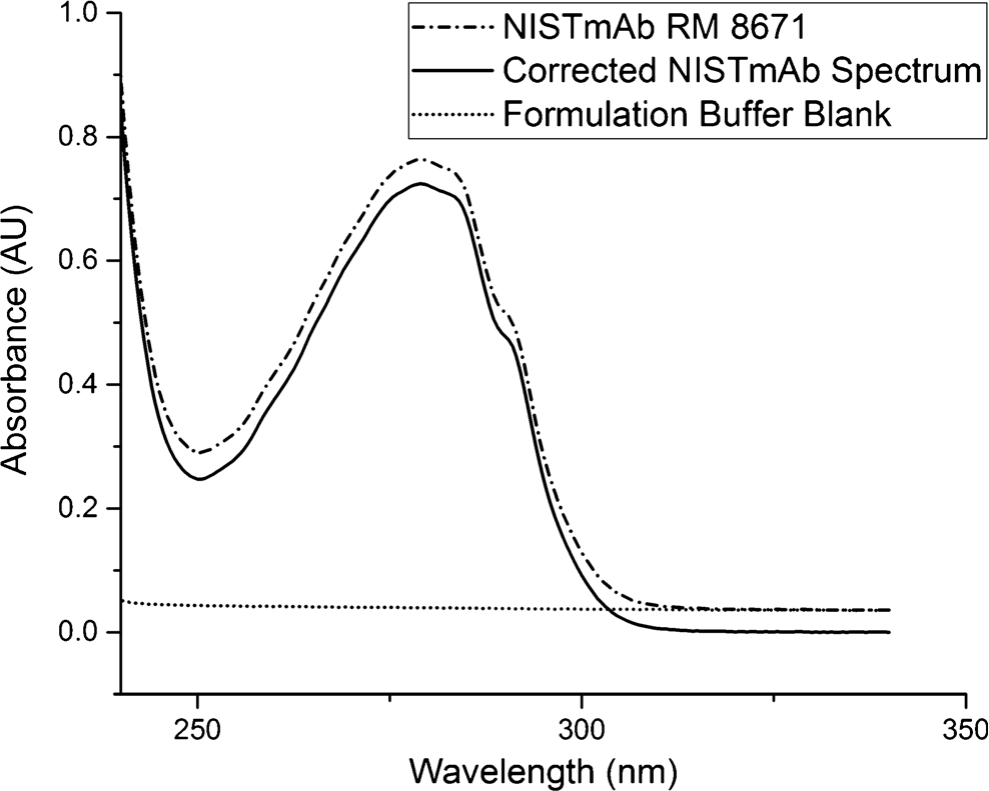
Representative spectra obtained for the Formulation Buffer blank (∙∙∙∙∙∙), NISTmAb RM 8671 14HB-D-002 (∙ − ∙ − ∙ − ∙), and the blank-subtracted (corrected) NISTmAb spectrum (───)

Representative spectra for the Formulation Buffer (∙∙∙∙∙∙) and NISTmAb RM 8671 (∙ − ∙ − ∙ − ∙) can be seen in Fig. 1. Figure 1 also depicts a representative blank-subtracted (corrected) NISTmAb spectrum (───). This spectrum displays decadic attenuance characteristic of a protein, with an apex of absorption at ≈280 nm.

The corrected decadic attenuance at 280 nm (*D*_*corr*_) for each sample was calculated by subtracting the mean D280 blank measurement from each sample D280 measurement. Intra-vial measurement repeatability was evaluated by performing triplicate analyses from the same vial of lot 14HB-D-001 and resulted in a Coefficient of Variation (CV) of 0.065% (based on the standard deviation of the measured decadic attenuance, *SD*_*D*_). The inter-vial homogeneity (*n* = 10 vials per lot) resulted in a CV of 0.090%, 0.176%, and 0.137% (based on *SD*_*D*_ for lots D-001, D-002, and D-003 respectively); only slightly larger than the intra-vial precision. There was no apparent trend in the data with respect to vial/rack position within a lot or when plotted against the sequence in which the samples were prepared. Similar results were found for each of the three lots, indicating the vial filling process was homogeneous across all racks from an individual lot.

Reference decadic attenuance values determined for each lot of NISTmAb are given in Table S2 in the ESM. The combined standard uncertainty associated with the *D*_*corr*_ measurement includes multiple contributions as described in the ESM [14, 15]. Metrological traceability is to the decadic logarithm of the derived unit of regular spectral transmittance through the NIST Transfer Spectrophotometer (TS), which is qualified against the HAS II National Spectrophotometer via control standard SRM 2031 at 280 nm. The *D*_*corr*_ reference values reflect measurements conducted using the specific quartz cuvette (*b* = 0.05092 cm). In an effort to report a more universal reference value amenable to direct comparison with other spectrophotometric determinations (e.g. alternative path length and/or dilutions), *D*_*corr*_ was further used along with Eq. 1 to calculate reference mass concentration values reported in Table S3 (see ESM).

**Fig. 2:**
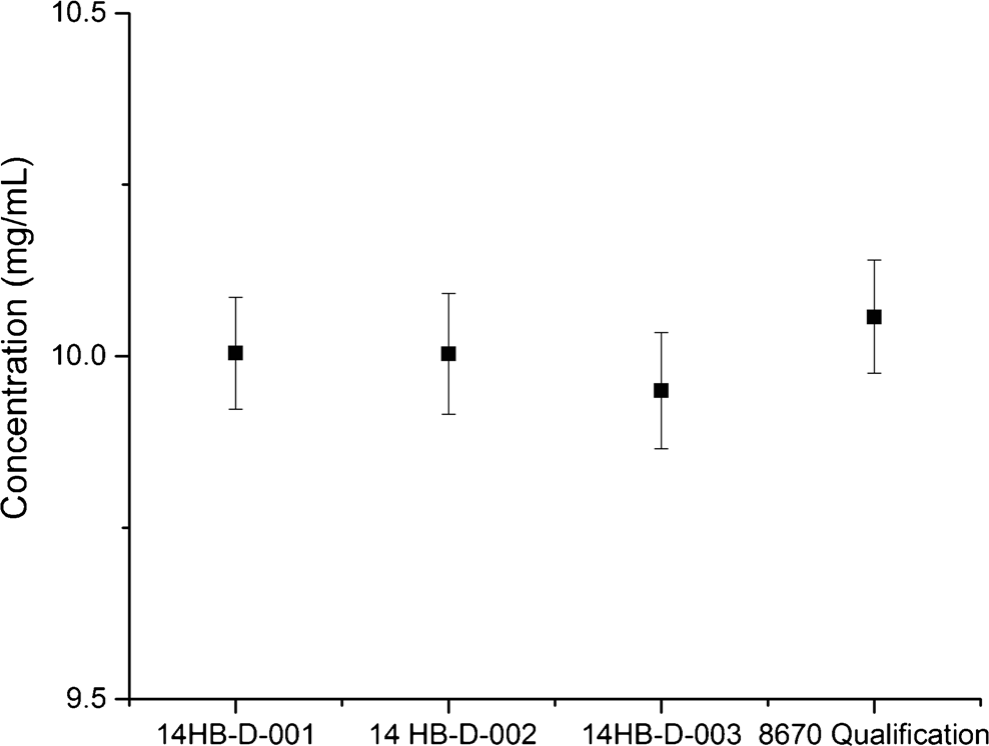
Mean concentration determined for each lot of NISTmAb using UV-Vis spectrophotometry. Error bars represent ±2*u*_*c*_ based on statistical treatment of data as described in ESM (*n* = 10 for RM 8671 lots, *n* = 2 for PS 8670)

The reference values listed in Table S3 (see ESM) and Fig. 2 are based specifically on measured decadic total attenuance at 280 nm assuming a theoretical extinction coefficient (*ε*) of 1.42 (mL mg^−1^ cm^−1^) [16]. The extinction coefficient was calculated according to the method reported by Pace et al. [19], and further corrected for glycan mass fraction via a correction factor of 0.977 [16]. Uncertainty associated with the theoretical extinction coefficient has not been fully evaluated; therefore the reported value is not traceable to the SI unit of mass. A comprehensive uncertainty budget is reported in the ESM such that a future experimental determination of ε may be incorporated if deemed necessary via stakeholder feedback. The combined standard uncertainty provided in Table S3 (see ESM) is intended to represent, at the level of one standard deviation, the effect of the combined components of uncertainty including Type A measurement uncertainty and Type B components related to the analysis, consistent with the International Standards Organization/Joint Committee for Guides in Metrology (ISO/JCGM) Guide [20]. Each of the three 14HB-D lots were measured to have a concentration to within ±2*u*_*c*_ of one another (Fig. 2) which demonstrates that good inter-lot reproducibility was achieved. These data indicate the lifecycle management plan using bulk homogenization produced highly reproducible lots, and therefore future lots of RM 8671 are expected to be consistent with respect to concentration.

### Physicochemical reference values

#### Physicochemical methods: Capillary zone electrophoresis (CZE)

**Fig. 3:**
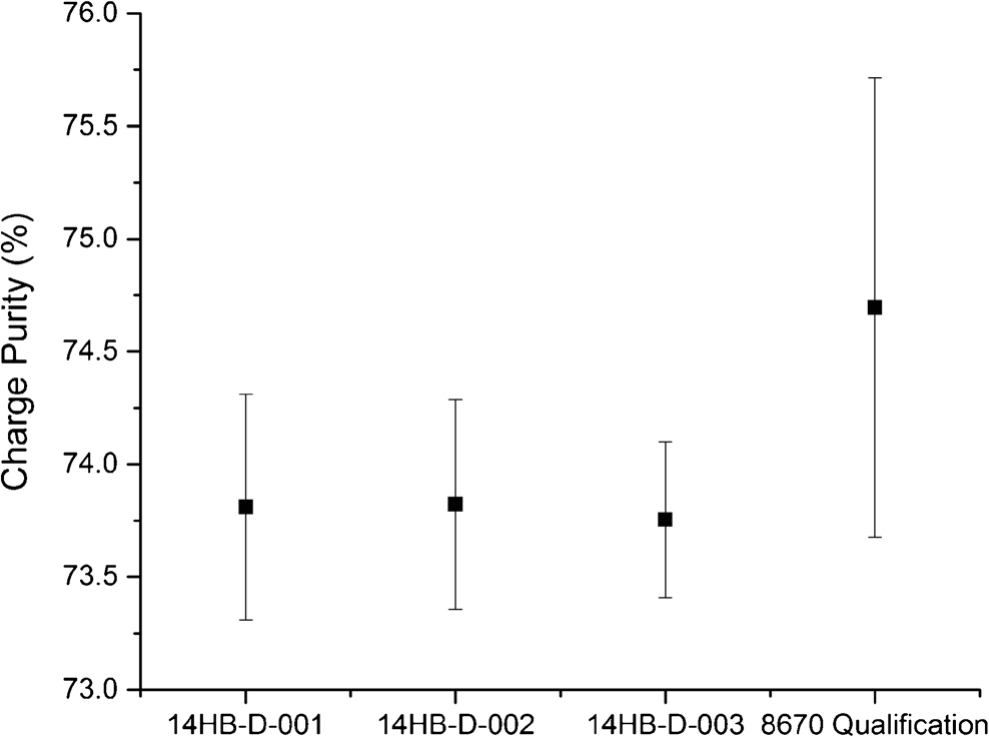
Homogeneity of RM 8671 lots by charge purity measured by CZE. Error bars represent ±3*u*_*c*_ based on ANOVA analysis for each lot (*n* = 3 for RM 8671 lots) or from intermediate precision data for PS 8670 Qualification [11]

NISTmAb charge heterogeneity was evaluated by the qualified CZE assay [12], wherein mAb charge variants are separated according to differential electrophoretic mobility in free solution within a uniform electric field applied across a buffer-filled fused silica capillary. The results of the CZE analysis of RM 8671 were consistent in all salient features to those observed previously with PS 8670, having three charge groups: the main group, which comprises the majority of the sample; the basic variants, which migrate toward the cathode more rapidly than the main group; and the acidic variants, which migrate toward the cathode less rapidly than the main charge group [11]. The charge purity of the NISTmAb is given as the relative abundance of the main charge group with respect to all detected charge species. The results for RM 8671 compared to PS 8670 method qualification results are presented in Table S6 (see ESM) and Fig. 3.

The inter-vial homogeneity of the three lots of RM 8671 was assessed from the CZE data. The CV for main peak purity is less than 0.2% in each of the lots as calculated from the results listed in ESM Table S6. Standard deviation calculated for intra-vial variation compared to inter-vial variation on the raw data for main peak purity was also nearly identical (e.g. intra-vial *SD* = 0.109 vs. inter-vial *SD* = 0.131 for lot 14HB-D-001 main peak purity). Collectively this indicates inter-vial homogeneity with respect to charge purity was achieved.

The three lots of RM 8671 were found to conform to previously observed results for PS 8670 for all charge parameters whose values (ESM Table S6) agreed to within ±3*u*_*c*_, as shown in Fig. 3 for the main peak purity. The one-way two-tailed ANOVA, however, did reveal a minor difference from PS 8670. RM 8671 lots were found to contain slightly increased basic variants (≈1%) relative to PS 8670. This difference may be attributable to expected minor variations in the cellular expression/production of batches of material used for PS 8670 versus RM 8671. The three lots originating from the homogenized bulk (RM 8671), however, were shown to be consistent with one another in terms of main peak purity, basic variant relative abundance, and acidic peak relative abundance. Figure 3 demonstrates that good inter-lot reproducibility was achieved for each of the three 14HB-D lots, and that each material was observed to have main peak purity to within ±3*u*_*c*_ of one another. Despite a small decrease in apparent charge purity versus PS 8670, it is the consistency of the commercial (RM 8671) lots that are important in ensuring a consistent product to stakeholders, which the current data indicate will be true considering the statistical equivalence of all three commercial lots. The current data suggest that the bulk homogenization method used to produce the RM 8671 lots was successful in homogenizing any minor process related variability (e.g. C-terminal lysine occupancy) and produced highly consistent, reproducible lots, thereby ensuring long term consistency in product quality attributes.

#### Physicochemical methods: Size exclusion chromatography (SEC)

**Fig. 4:**
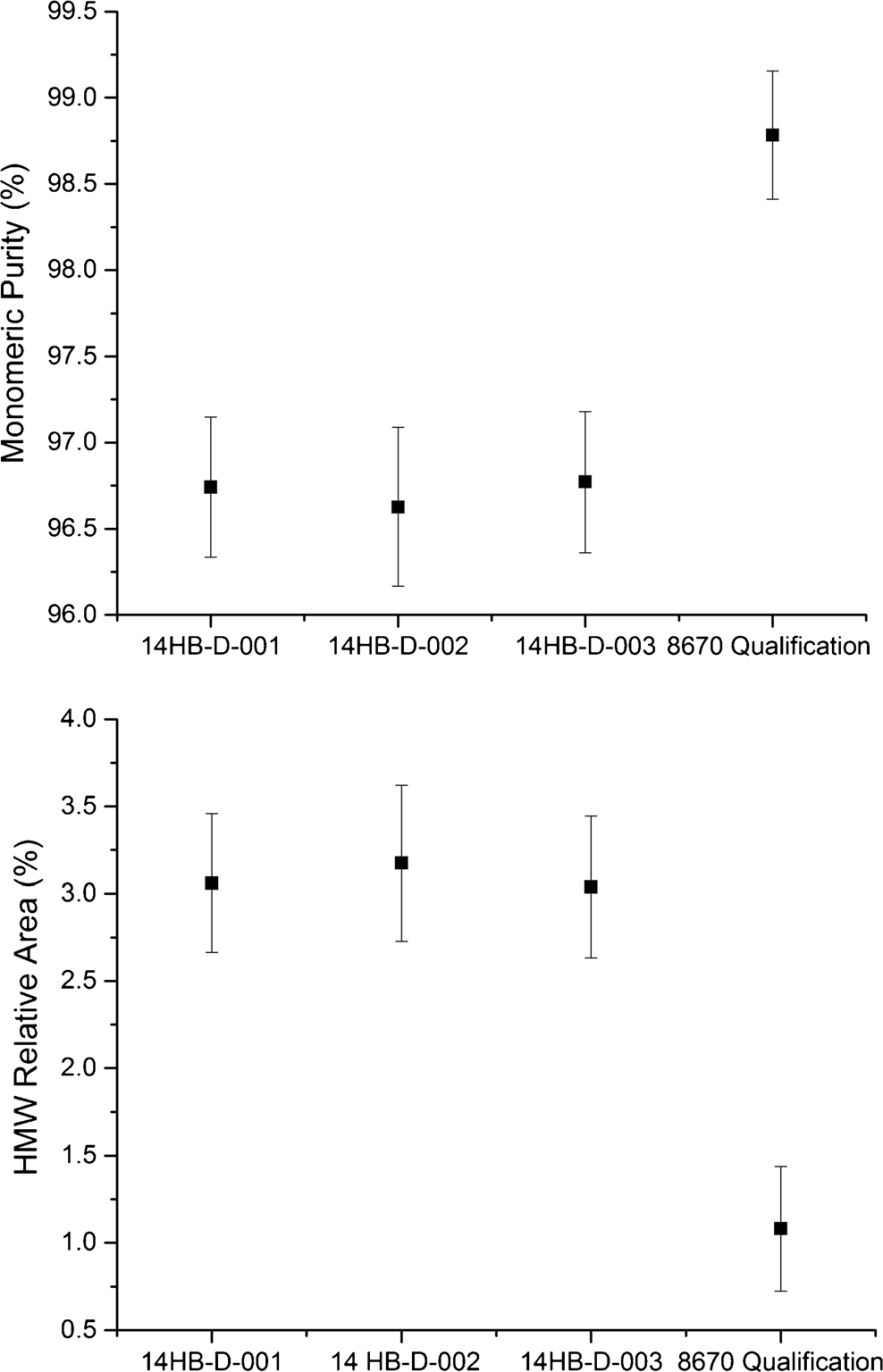
Homogeneity of RM 8671 lots by monomeric purity (top panel) and high molecular weight relative area (bottom panel), measured by SEC. Error bars represent ±3*u*_*c*_ based on ANOVA analysis for each lot (*n* = 3 for RM 8671 lots) or from intermediate precision data for PS 8670 Qualification [12]

The size heterogeneity and monomeric purity of NISTmAb RM 8671 were analyzed under non-denaturing conditions by SEC with UV detection according to the qualified protocol [12]. The resultant chromatograms for all three RM 8671 lots were consistent in all salient features to that observed previously with PS 8670. The parameters considered were monomeric purity (main peak relative area), high molecular weight (HMW) relative area (RA), and low molecular weight (LMW) RA. Figure 4 and Table S7 (see ESM) present results for PS 8670 during method qualification [12] compared to the results during value assignment for RM 8671. The CV for monomeric purity is approximately 0.1% in each of the individual lots as calculated from the results listed in ESM Table S7. Standard deviation calculated for intra-vial replicates compared to inter-vial variation on the raw data for main peak purity was also nearly identical (e.g. intra-vial SD = 0.049 vs. inter-vial SD = 0.049 for lot 14HB-D-001 monomeric purity). Collectively this indicates inter-vial homogeneity with respect to monomeric purity was achieved for each individual lot.

It is clear from Fig. 4 that RM 8671 does not conform to the values determined for PS 8670. The relative area of the monomer has decreased from ≈98.7% in PS 8670 to ≈96.7% in RM 8671 and the relative area of the high molecular weight species has increased from ≈1% to ≈3% (ESM Table S7). Despite a small increase in the % HMW versus PS 8670, the three lots of RM 8671 were shown to be consistent with one another in terms of monomeric purity, high molecular weight relative area and low molecular weight relative area. Figure 4 demonstrates that good inter-lot reproducibility was achieved for each of the three 14HB-D lots, and that each material was observed to contain % monomer, % HMW, and % LMW to within ±3*u*_*c*_ of one another. Alterations in the homogenization and vial filling process and/or an increased presence of HMW species in one of the constituent batches, versus the PS 8670 material, are thought to have resulted in the increased HMW species. Despite a small increase in the % HMW versus PS 8670, it is the inter-lot homogeneity of the commercial (RM 8671) lots that is important in ensuring a consistent product to stakeholders, which the current data indicate will be true considering the statistical equivalence of all three commercial lots with respect to aggregates based on SEC.

#### Physicochemical methods: Capillary electrophoresis-sodium dodecyl sulfate (CE-SDS)

**Fig. 5:**
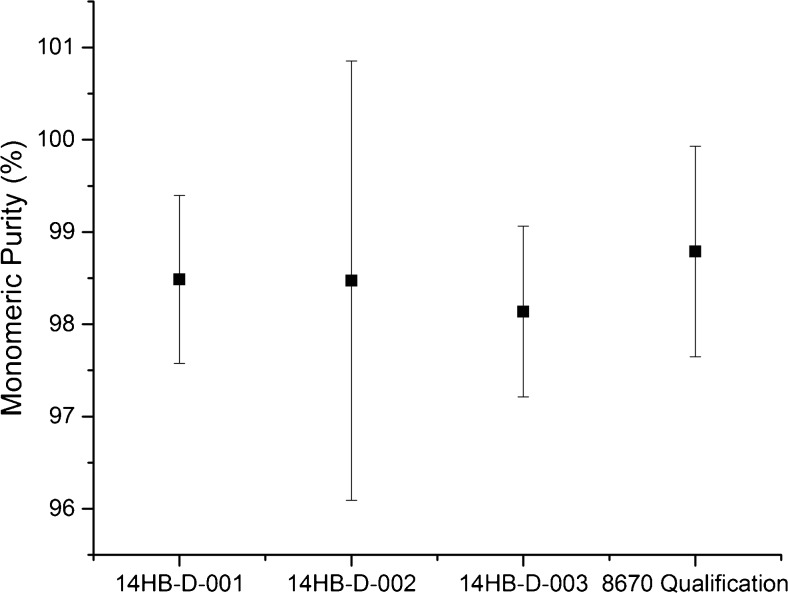
Homogeneity of RM 8671 lots by monomeric purity, measured by nrCE-SDS. The measured range for PS 8670 during qualification is included for comparison. Error bars represent ±3*u*_*c*_ based on ANOVA analysis for each lot (*n* = 3 for RM 8671 lots) or from intermediate precision data for PS 8670 Qualification [12]

CE-SDS is the micro electrophoretic analogue of traditional slab-gel size-based separations (e.g. SDS-PAGE). Analytes are complexed with SDS and injected into a narrow-bore glass capillary filled with an uncrosslinked polymer sieving matrix. A high voltage applied across the capillary drives the anionic SDS complexes toward the detection window; their migration is retarded in a size-dependent manner by differential interaction with the sieving matrix. NISTmAb RM 8671 monomeric purity was measured by CE-SDS under non-reducing conditions (nrCE-SDS) according to the qualified method protocol [12]. Glycan occupancies of the heavy chain and relative abundance of non-reducible species were measured by CE-SDS under reducing conditions (rCE-SDS) according to the qualified method protocol [12]. The electropherograms of the CE-SDS analyses of RM 8671 were consistent in all salient features to that observed previously with PS 8670 [12]. The results for RM 8671 compared to PS 8670 method qualification results are presented in Table S8 (see ESM) and Fig. 5. In addition to the calculated physicochemical reference values listed in ESM Table S8, it was determined that method performance (migration times and relative abundance) of individual, resolved components (monomer, light chain, heavy chain, etc.) may be useful to the stakeholder in comparing performance of orthogonal assays likely to be developed with the NISTmAb. Therefore additional Tables S9, S10, S11, and S12 are included in the ESM for PS 8670 (from the qualification exercise) and RM 8671 lots 14HB-D-001, 14HB-D-002, and 14HB-D-003 respectively.

Inter-vial homogeneity of the three lots of RM 8671 was also evaluated for each lot using CE-SDS. The CV for monomeric purity ranges from 0.3% to 0.8% calculated from the results listed in ESM Table S8. Standard deviation calculated for intra-vial variation compared to inter-vial variation on the raw data for main peak purity was also similar (e.g. intra-vial SD = 0.053 vs. inter-vial SD = 0.137 for lot 14HB-D-001 monomeric purity). An F-test for equivalence of variance confirmed the standard uncertainties were statistically similar. Collectively these indicate inter-vial homogeneity with respect to monomeric purity was achieved.

The three lots of RM 8671 were found to conform to previously observed results for PS 8670 for all size parameters measured to within the ±3*u*_*c*_ control range listed in ESM Table S8. The one-way two-tailed ANOVA, however, did reveal a minor (nearly negligible, <0.03%) difference from PS 8670 in the glycan occupancy. This difference may be attributable to expected minor variations in the cellular expression/production of batches of material used for PS 8670 versus RM 8671.

All three lots of RM 8671 were shown to be consistent with one another in terms of monomeric purity, thioether content, and glycan occupancy demonstrating good inter-lot homogeneity for these properties (ESM Table S8). Figure 5, for example, demonstrates that each material was observed to contain monomeric purity within ±3*u*_*c*_ of one another. The current data suggest that the bulk homogenization method used to produce the RM 8671 lots was successful in producing highly consistent, reproducible lots, thereby ensuring long term consistency in product quality attributes.

### Informational values

#### Flow imaging

**Fig. 6:**
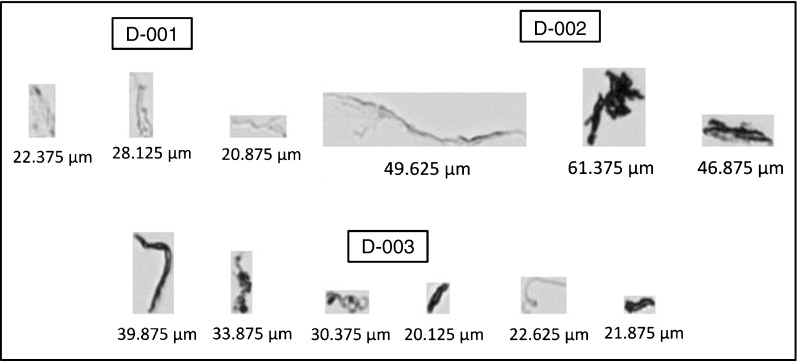
Representative images of proteinaceous particles and their sizes (in equivalent circular diameter) obtained from 14HB-D-001, 14HB-D-002, and 14HB-D-003 analyses

Flow imaging can be used for the analysis of subvisible particles (2 μm to 100 μm in size) in suspension. As a sample stream passes through a flow cell positioned in the field of view of a microscopic system, bright-field images are captured in successive frames. The digital images of the particles present in the sample are stored in a database that can be retrieved and analyzed for count, size, transparency, and various other morphological parameters. Protein particle concentration is reported for one or more size bins as an equivalent circular diameter (ECD), defined as the diameter of a polystyrene microsphere with the same image area as the observed particle. The subvisible protein particle content of RM 8671 lots was evaluated according to the optimized protocol described previously [12]. Figure 6 shows representative images obtained for various particles in the three lots.

Table S13 (see ESM) shows the mean subvisible particle concentrations (ECD ≥ 2 μm) in the PS 8670 and the three lots of RM 8671. Each of the vials of PS 8670 was obtained from the same rack of material, such that vial to vial variability due to the fill process can be assumed to be negligible. The RM 8671 vials, on the other hand, were obtained from multiple racks across the fill sequence. The CV (as calculated from ESM Table S13) for the inter-vial variability of each of the RM 8671 lots is on the same order as observed for PS 8670, indicating little to no vial-to-vial heterogeneity with regard to protein particle content was introduced by the filling process. From Table S13 (see ESM), the variability appears to be high even between vials in a particular lot; however, no clear trend among the racks from which the samples were drawn was observed. Due to the fragile nature of protein particles and the sensitivity of particle production to small changes in vial storage or handling, higher variability in particle concentration is expected. It should be noted that even if the concentration of particles were dissimilar from one vial to another, the amount of protein present in the particles is very minute, on the order of tens to several hundreds of ng/mL, corresponding to <0.005% protein concentration in solution [12].

All three lots of RM 8671 were shown to be consistent with one another, as well as to the PS 8670 material with respect to total particle content (within ±3SD). This indicates that the fill finish sequence did not significantly introduce particulates in the size range ≥ 2 μm and that consistent production of future lots can be expected. Table S13 does, however, show a potential trend of increasing particle content with increasing lot number. Trending of additional lots of material may be useful in the future to discern if this is indeed a trend or within typical lot-to-lot variability.

#### Dynamic light scattering

To obtain a size distribution of particles ranging from 1 nm to 1 μm in size, dynamic light scattering (DLS) was used. Particles in a solution move randomly by Brownian motion and scatter light. By analyzing the fluctuations in the intensity of the scattered light as a function of time, the diffusion coefficient of the particles and consequently their size can be calculated. The hydrodynamic diameter of RM 8671 lots was evaluated according to the optimized protocol described previously [12]. Table S14 (see ESM) shows the mean hydrodynamic diameters of the samples from the three lots, which ranged from 9.83 nm to 9.96 nm.

All three lots of RM 8671 were shown to be consistent with one another, as well as to the PS 8670 material with respect to the mean hydrodynamic radius (within ±3SD). Each of the three lots also showed similar particle profiles in the nanometer range supporting the assertion that the vial filling process for the RM 8671 material appears to be homogeneous across all racks.

### Identity

#### LC-UV-MS/MS peptide map

The peptide mapping method examines RM 8671 primary structure by monitoring its trypsin digested peptides, which are resolved using reverse-phase ultrahigh pressure liquid chromatography instrumentation coupled to an ultraviolet wavelength detector and a high-resolution mass spectrometer with electrospray ionization source (together “LC-UV-MS/MS”). A chromatographic trace, or peptide map, results from the signal generated as peptides eluting from the LC column pass through the UV and MS detectors producing “peaks”. Differing amino acid sequences give each peptide unique chromatographic properties and the presence of online MS/MS detection provides confident assignment of primary structure based on mass and fragmentation consistent with the predicted amino acid sequence.

**Fig. 7:**
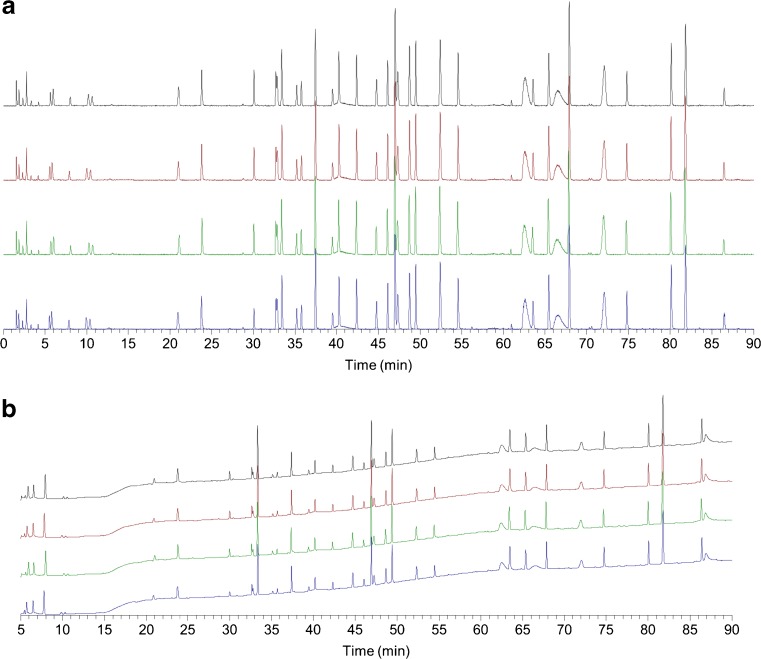
Alignment of PS 8670 chromatogram with RM 8671 lots 14-HB-D-001, 14-HB-D-002, and 14HB-D-003 chromatograms. Tryptic digests of PS 8670 and RM 8671 lots 14-HB-D-001, 14-HB-D-002, and14HB-D-003 (traces ordered top to bottom) were analyzed by LC-UV-MS and the similarity of the resulting (**A**) TIC and (**B**) UV chromatograms compared against the reference peptide map generated from the PS 8670 digest (top, black trace in panels a and b). The initial five minutes of the UV traces are not shown due to the large difference in scale between the relative levels of absorbance of peaks detected during the 0 min to 5 min period and the 5 min to 90 min period

LC-UV-MS/MS peptide mapping was performed on all three lots of RM 8671 in parallel with PS 8670 according to the optimized protocol described previously [9]. Alignment of the TIC and UV traces of the RM 8671 digest with the PS 8670 reference peptide map showed a high degree of similarity upon visual inspection (Fig. 7). No trace had a unique or missing peak as compared to the reference map.

Mean TIC retention times were calculated across quadruplicate injections of the PS 8670 digest and data from three of the injections were used to calculate mean UV retention times. The difference between means of the PS 8670 reference map peak retention times and the corresponding peaks for the three lots of RM 8671 was <2% for all peaks in the TIC and UV chromatograms, indicating a high degree of similarity between PS 8670 and the RM 8671 lots.

To further confirm the identity of NISTmAb RM 8671, data from its tryptic digest were submitted for peptide identification. Calculation of the sequence coverage for each tryptic digest produced the same results as those described previously for PS 8670 and lot 14HB-D-001 [9]. For all materials analyzed, sequence coverage of 96.89% was achieved for the heavy chain and 100% for the light chain. This included full coverage of the complementarity-determining regions (CDRs). All post translational modifications identified for the three RM 8671 lots were consistent with those previously reported for PS 8670 [9]. The mass spectrometry results indicated nearly complete pyroglutamination of the N-terminus, moderate levels of the loss of C-terminal lysine, and low levels of glycation, oxidation, and deamidation as previously reported [9]. The similarity of the TIC and UV peptide maps of PS 8670 and RM 8671 as well as the matching peptide identifications confirmed the identity of RM 8671 as conforming to PS 8670.

### Stability

Each vial of RM 8671 contains 800 μL of a nominal 10 mg/mL IgG1κ monoclonal antibody (NISTmAb) in 12.5 mmol/L L-histidine, 12.5 mmol/L L-histidine -HCl- (pH 6.0) (Formulation Buffer). RM 8671 is packaged and supplied to the user in internal threaded polypropylene cryovials which have been double packaged in a cardboard box with an insert designed to securely fit the cryovials, and sealed in a laminate foil pouch to prevent CO_2_ ingress. The double packaged RM is shipped on dry ice and should remain frozen during shipment. The material should be stored in a frozen state at −80 °C immediately upon receipt and in all cases storage of the material at −80 °C is preferred. In reality, no fill volume/format will be amenable to all assays and end user purposes. Therefore, a series of stability samples (preparation described in ESM) were evaluated to determine the most extreme storage conditions (referred to here as alternative storage conditions) under which the sample yielded a measurement result within ±3*u*_*c*_ for a given method.

**Fig. 8:**
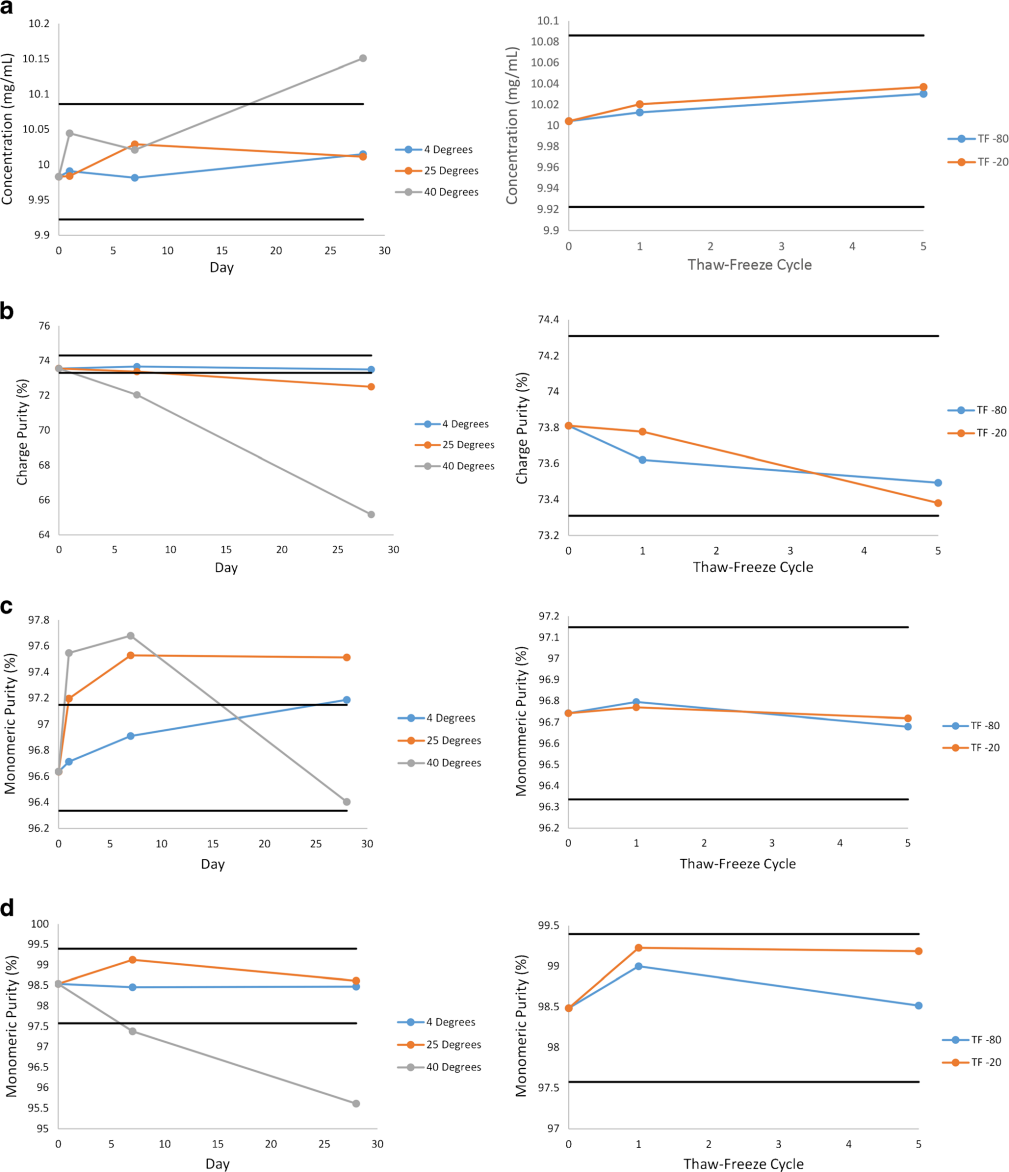
Physicochemical method results for 14HB-D-001 under accelerated stablity (left) and thaw/freeze conditions using (**a**) UV-Vis, (**b**) CZE, (**c**) SEC, and (**d**) nrCE-SDS. The black lines indicate a control range taken as the mean value of unstressed 14HB-D-001 ± 2*u*_*c*_ for UV-Vis and ±3*u*_*c*_ for physicochemical assays

Storage stability evaluation was performed on RM 8671 lot 14HB-D-001 and extrapolated for all other RM 8671 lots. Samples reserved for thaw/freeze stability and accelerated stability analyses were reserved as indicated in the ESM (see Table S15 and Table S16). T/F samples were tested using the qualified physicochemical assays or optimized informational value assays (at a minimum) described above after undergoing an additional one to five T/F cycles with freeze temperature at −80 °C or −20 °C. Accelerated stability samples were analyzed using the qualified physicochemical assays or optimized informational value assays described above at the (0, 7, and 28) day time points described in the ESM (see Table S16). The one day time points were also analyzed with SEC to provide more fine-detail in excursions from the control limits at elevated temperatures. Control charts for alternative storage stability evaluations displaying measurement results for the various stability conditions, with control range taken as the mean value of the unstressed material (±2*u*_*c*_ for UV and ±3*u*_*c*_ for physicochemical assays), are depicted in Fig. 8. A larger control range of ±3*u*_*c*_ was utilized for physicochemical assays compared to the concentration measurement because of the fewer degrees of freedom and increased measurement variability observed during value assignment. In addition, the UV concentration measurement is expected to be less sensitive to degradation, yet deviations in concentration may have significant impact on other physicochemical measurements.

**Fig. 9:**
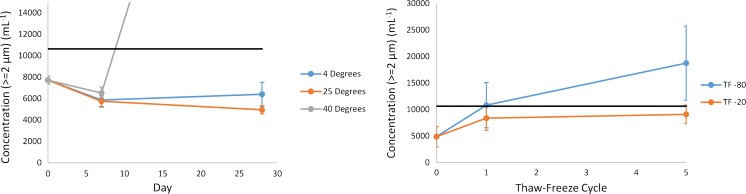
Mean particle concentration (ECD ≥ 2 μm) results for 14HB-D-001 under accelerated stablity (left) and thaw/freeze (right) conditions using flow imaging. The black line indicates the upper control limit taken as the mean value of unstressed 14HB-D-001 + 3*SD* and the error bars represent ±1*SD* for the specific sample. Note that the 40 °C 28 day sample (54,627 ml^−1^) is indicated with a straight line extending off of the graph

**Fig. 10:**
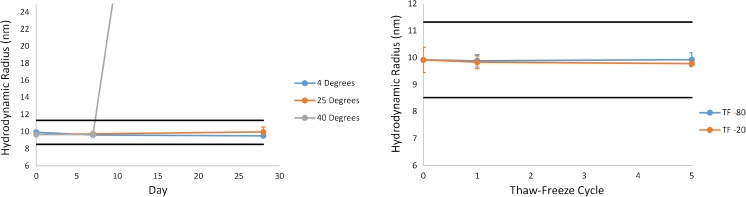
Mean hydrodynamic diameter results for 14HB-D-001 under accelerated stablity (left) and thaw/freeze (right) conditions using DLS. The black lines indicate the control range taken as the mean value of unstressed 14HB-D-001 ± 3*SD* and the error bars represent ±1*SD* for the specific sample. Note that the 40 °C 28 day sample (143 nm) is indicated with a straight line extending off of the graph

Control charts for alternative storage stability evaluations with respect to informational value properties are displayed in Fig. 9 and Fig. 10. Informational value measurement results utilized a stability control range taken as the mean value of the unstressed material ±3*SD*.

Recommended maximum storage T/F cycles and maximum storage time at a given temperature were set (for each method individually) as the most extreme data point that remained within the control range. NISTmAb RM 8671 was shown to be quite stable with respect to T/F according to UV, SEC, CE-SDS, and CZE. In all cases the material produced a measurement result within the control range for up to 5 T/F cycles. According to Fig. 9, however, the T/F conditions appeared to show particle concentrations near or outside of the defined control regions, therefore it is recommended that T/F not be performed for samples intended for flow imaging analysis.

Elevated storage temperature and time did affect the measurement results in some cases, as expected. Incubation at 40 °C for extended periods produced the most noticeable change, with an increase in protein particulate content, apparent hydrodynamic radius, and apparent concentration. SEC results at 40 °C indicated an initial decrease in HMW species, followed by an increase in LMW species beyond 7 days, resulting in the parabolic behavior of Fig. 8C under this condition. The kinetics were slowed under refrigerated conditions, however, SEC results did fall outside of the control range when stored at 4 °C for 28 days. Measurement results for all other assays were shown to be within the control limits during refrigerated storage for up to 28 days. In all cases, storage of the material at −80 °C is preferred. If aliquot preparation and/or storage at other than recommended conditions are necessary, the maximum alternative storage condition evaluated that yielded measurement results within the control range for each method is listed in ESM Table S17 for reference values and ESM Table S18 for informational values and peptide mapping.

## Discussion

A comprehensive lifecycle management program including a two-tiered in-house reference standard approach was modeled after industry best practices [10]. In order to serve as a common framework for pre-competitive innovation, a Reference Material must be shown to be homogeneous, stable, and fit for its intended use. The current manuscript describes in detail the stratified sampling plan that was used in association with rigorously qualified analytical methods to assign reference values and ensure the quality of the NISTmAb RM 8671 [9, 11, 12]. Reference value assignment was performed in concert with the in-house primary sample PS 8670 as a system suitability control, the material for which the most historical analytical and biophysical attribute information was available [6–8]. The initial methods selected were intended to be those that were most pertinent to the stakeholder in ensuring a quality material, and thus included chemical and colloidal stability/purity indicating assays along with a comprehensive primary amino acid sequence confirmation.

The homogeneity assessment of each individual lot was made at the time value assignment analyses were performed using CZE, SEC, CE-SDS, FI, and DLS. There was no apparent trend in the data with respect to vial/rack position within a lot or when plotted against the sequence in which the samples were prepared for any of the methods. Furthermore, comparison of each of the initial three lots of RM 8671 demonstrated statistical equivalence in all of the attributes measured. Although each lot of RM 8671 is assigned its own individual reference values, the intra- and inter-lot homogeneity indicates a successful homogenization and vial filling process resulting in consistent content and quality. It is therefore expected that consistent measurement results will be attainable for RM 8671 for the foreseeable future provided the samples are handled upon receipt as directed.

Stability with respect to physicochemical attributes was evaluated based on extended thaw/freeze testing and storage at various temperatures. In all cases, except for particle generation with multiple T/F cycles, the ability of the material to withstand several T/F cycles and extended storage at 4 °C (as indicated in Table S17 and Table S18 in the ESM) provides users a variety of options to suit their individual testing needs. Optimal storage for extended periods is in a frozen state at −80 °C. Prior to analysis, the vial should be removed from the −80 °C freezer and thawed at room temperature for approximately 30 min or until no residual ice crystals remain. Once thawed, the vial should be gently inverted five times to alleviate any concentration gradients that may have formed during the freezing process. The vial should then be briefly spun in a mini-centrifuge to settle any solution that may otherwise remain adhered to the lid or internal threads of the vial. When handled according to the optimum storage conditions, RM 8671 is expected to result in physicochemical performance within the assigned control range (ESM Table S17) for each qualified physicochemical reference value method described previously in this series [11, 12].

RM 8671 has demonstrated stability and homogeneity with respect to the specified physicochemical properties using the qualified methods described herein, and is consequently suitable as a Reference Material. Significant effort was made to ensure lot-to-lot homogeneity and stringent method optimization to produce measurements with minimal sample preparation artifacts. Ultimately the values, from a metrological perspective, are method-specific and intended as a baseline for comparison of results from orthogonal, yet related assays that may be performed in a given user’s laboratory. This measurement approach, however, is indeed fit-for-purpose as the measurement technologies selected are representative of the current state-of-the-art in biopharmaceutical characterization. The availability of a Reference Material along with the well-defined analysis parameters reported throughout this publication series provides for the first time a foundation upon which longitudinal advances in biopharmaceutical characterization can be benchmarked. Future measurement technologies, regardless of their mechanistic principles, can be traced back to a consistent quality material of the same constituent product quality attributes.

Advances in technology are inevitable, and will ultimately be the future of higher-resolution product understanding. The pre-competitive nature of NISTmAb RM 8671 makes it a useful, open-innovation platform to demonstrate novel analytical and biophysical technologies and/or competencies within an organization. It affords a common platform for discussion, harmonization and advancement of analytical and biophysical technologies as may be achieved through inter-laboratory comparisons. The presence of a widely available industry quality material, analyzed by countless permutations of every analytical tool available, will produce the most expansive and dynamic single protein characterization dataset to date. Continued operation in the pre-competitive space offers an unprecedented opportunity to harness these big data for developing foundational tools such as complex inter-method data integration, modeling, and data visualization to guide next-generation biopharmaceutical development.

## Conclusions

Enactment of the NISTmAb RM 8671 quality plan is described herein, demonstrating identity, quality, and stability of this material with respect to its material properties. The NISTmAb represents a first-of-its-kind Reference Material intended for use in evaluating measurement technologies to inform on specific attributes during therapeutic protein characterization. With this unique role in mind, a novel lifecycle management plan was developed including a two-tier in-house reference standard system, industry-relevant concentration measurement, qualified physicochemical assays, and relevant informational measurements. RM 8671 was verified to be homogeneous both within and between vialing lots, demonstrating the robustness of the lifecycle management plan. It was analyzed in concert with the in-house primary sample PS 8670 to provide a historical link to this seminal material. RM 8671 was verified to be fit for its intended purpose as a technology innovation tool, external system suitability control, and cross-industry harmonization platform. The pre-competitive nature and long term availability of RM 8671 should provide a platform for monitoring the evolution of therapeutic protein characterization.

## Electronic supplementary material

ESM 1 (PDF 2.01 MB)

## References

[1] International Counsil for Harmonisation. Q6A: Specifications: test procedures and acceptance criteria for new drug substances and new drug products: chemical substances. ICH Harmonised Tripartite Guideline; 1999. http://www.ich.org/fileadmin/Public_Web_Site/ICH_Products/Guidelines/Quality/Q6A/Step4/Q6Astep4.pdf.

[2] International Counsil for Harmonisation. Q6B: Specifications: test procedures and acceptance criteria for biotechnological products. ICH Harmonised Tripartite Guideline; 1999. http://www.ich.org/fileadmin/Public_Web_Site/ICH_Products/Guidelines/Quality/Q6B/Step4/Q6B_Guideline.pdf.

[3] Arbogast LW, Brinson RG, Formolo T, Hoopes JT, Marino JP (2016). 2D (1)H(N), (15)N correlated NMR methods at natural abundance for obtaining structural maps and statistical comparability of monoclonal antibodies. Pharm Res.

[4] Arbogast LW, Brinson RG, Marino JP (2015). Mapping monoclonal antibody structure by 2D 13C NMR at natural abundance. Anal Chem.

[5] Arbogast LW, Brinson RG, Marino JP (2016). Application of natural isotopic abundance (1)H-(1)(3)C- and (1)H-(1)(5)N-correlated two-dimensional NMR for evaluation of the structure of protein therapeutics. Methods Enzymol.

[6] Schiel JE, Davis DL, Borisov OB, editors. State-of-the-art and emerging technologies for therapeutic monoclonal antibody characterization, volume 1. Monoclonal antibody therapeutics: structure, function, and regulatory space. ACS Symposium Series, vol 1176. Washington, DC: American Chemical Society; 2014.

[7] Schiel JE, Davis DL, Borisov OB, editors. State-of-the-art and emerging technologies for therapeutic monoclonal antibody characterization, volume 3. Defining the next generation of analytical and biophysical techniques. ACS Symposium Series, vol 1202. Washington, DC: American Chemical Society; 2015a.

[8] Schiel JE, Davis DL, Borisov OB, editors. State-of-the-art and emerging technologies for therapeutic monoclonal antibody characterization, volume 2. Biopharmaceutical characterization: the NISTmAb case study. ACS Symposium Series, vol 1201. Washington, DC: American Chemical Society; 2015b.

[9] Mouchahoir T, Schiel JE (2018). Development of an LC-MS/MS peptide mapping protocol for the NISTmAb. Anal Bioanal Chem.

[10] Schiel JE, Turner A (2018). The NISTmAb Reference Material 8671 lifecycle management and quality plan. Anal Bioanal Chem.

[11] Turner A, Schiel JE (2018). Qualification of NISTmAb charge heterogeneity control assays. Anal Bioanal Chem.

[12] Turner A, Yandrofski K, Telikepalli S, King J, Heckert A, Filliben J, Ripple D, Schiel J (2018). Development of orthogonal NISTmAb size heterogeneity control methods. Anal Bioanal Chem.

[13] May W, Parris R, Beck C, Fassett J, Greenberg R, Guenther F (2000). Definitions of terms and modes used at NIST for value-assignment of reference materials. Special publication 260–136.

[14] Magnusson B, Ellison SL (2008). Treatment of uncorrected measurement bias in uncertainty estimation for chemical measurements. Anal Bioanal Chem.

[15] Travis JC, Smith MV, Choquette SJ, Liu H-K. NIST Technical Note 1715: certified transmittance density uncertainties for standard reference materials using a transfer spectrophotometer. 2011. http://ws680.nist.gov/publication/get_pdf.cfm?pub_id=908914.

[16] Gokarn Y, Agarwal S, Arthur K, Bepperling A, Day ES, Filoti D et al. Biophysical Techniques for Characterizing the Higher Order Structure and Interactions of Monoclonal Antibodies. State-of-the-Art and Emerging Technologies for Therapeutic Monoclonal Antibody Characterization Volume 2. Biopharmaceutical Characterization: The NISTmAb Case Study. ACS Symposium Series, vol 1201: American Chemical Society; 2015. p. 285–327.

[17] IUPAC. Compendium of Chemical Terminology, 2nd ed. (the “Gold Book”). Compiled by A. D. McNaught and A. Wilkinson. Oxford: Blackwell Scientific Publications; 1997. XML on-line corrected version: http://goldbook.iupac.org (2006) created by M. Nic, J. Jirat, B. Kosata; updates compiled by A. Jenkins. 10.1351/goldbook.

[18] Maity H, Wei A, Chen E, Haidar JN, Srivastava A, Goldstein J (2015). Comparison of predicted extinction coefficients of monoclonal antibodies with experimental values as measured by the Edelhoch method. Int J Biol Macromol.

[19] Pace CN, Vajdos F, Fee L, Grimsley G, Gray T (1995). How to measure and predict the molar absorption coefficient of a protein. Protein Sci.

[20] Joint Committee for Guides in Metrology. Evaluation of measurement data - guide to the expression of uncertainty in measurement (GUM 1995 with minor additions) 200:2008. https://www.bipm.org/en/publications/guides/gum.html.

