# Supplementary material for: The NISTmAb Reference Material 8671 value assignment, homogeneity, and stability
Source: Anal Bioanal Chem. 2018 Feb 7;410(8):2127–39. doi: 10.1007/s00216-017-0800-1 (PMC5830482; doi:10.1007/s00216-017-0800-1)
Supplement: Supplementary file 1 — (PDF 2.01 MB) [file 216_2017_800_MOESM1_ESM.pdf]

Analytical and Bioanalytical Chemistry

Electronic Supplementary Material

The NISTmAb Reference Material 8671 value assignment, homogeneity, and stability

John E. Schiel, Abby Turner, Trina Mouchahoir, Katharina Yandrofski, Srivalli Telikepalli, Jason King, Paul DeRose, Dean Ripple, Karen Phinney

SUPPLEMENTARY MATERIALS AND METHODS

Formulation Buffer. Formulation buffer (12.5 mmol/L L-His, 12.5 mmol/L L-His HCl, pH 6.0) was utilized as the blank solution for measurements. Formulation buffer was prepared as follows: 1) 1.3129 g histidine monohydrochloride monohydrate and 0.9704 g L-histidine were weighed out and diluted in a beaker with ~450 mL type 1 deionized ultrafiltered water (DIUF) that was obtained from an ultrapure water system fed with reverse osmosis water and filtered through a 0.2 µm filter.; 2) while recording pH with a calibrated pH meter, pH was adjusted by drop-wise addition of 1 mol/L hydrochloric acid to 6.00±0.02; 3) the solution was transferred to a 500 mL volumetric flask, the beaker rinsed with DIUF water and the flask volume adjusted to 500.0 mL using the rinse water; 4) the solution was sterile filtered into a sterile plastic bottle using a 0.22 µm cellulose acetate membrane; 5) the buffer was stored at 2 °C to 8 °C.

NISTmAb PS 8670 and RM 8671 Samples. Vialing of NISTmAb PS 8670 and RM 8671 (lots 14HB-D-001, 14HB-D-002, and 14HB-D-003) is described in the first publication of this series [1]. Samples reserved from each RM 8671 lot for homogeneity and value assignment were selected as indicated in Table S1 and Fig. S1 below. Thaw/freeze and accelerated stability samples were pulled sequentially from rack 1 of lot 14HB-D-001. Samples remained frozen during the reservation step.

Table S1 Vial Rack Diagram

Rack	UV Homogeneity	Physicochemical Homogeneity
**1**	1 random	3 same row (sequential)
**10**	1 random	
**20**	1 random	
**30**	1 random	
**40**	1 random	
**50**	1 random	3 same row (sequential)
**60**	1 random	
**70**	1 random	
**80**	1 random	
**90**	1 random	3 same row (sequential)

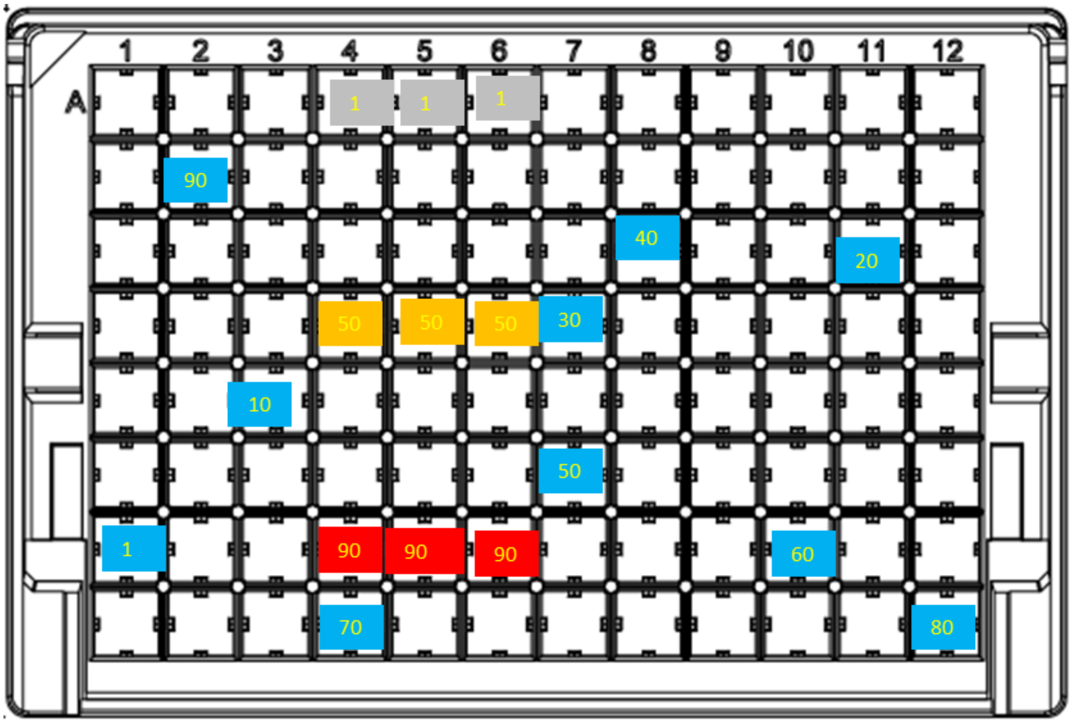

Fig. S1 Representative vial rack sampling pattern for value assignment and homogeneity assessment. Note actual positions were chosen at random. Numbers indicate rack number as sequentially unpacked and colors indicate the UV samples (Blue) and physicochemical reference value samples (gray, orange, and red), respectively

UV Optical Absorbance Value Assignment and Homogeneity Samples. Ten (10) samples from each lot were reserved for UV optical absorbance homogeneity and value assignment as depicted in Table S1 and Fig. S1. Vials from rows/columns were chosen at random from racks across the fill sequence. No further sample preparation was required for these samples. They were stored at −80 °C until use.

Statistical Treatment of UV Mass Concentration Reference Value. The blank-corrected decadic attenuance at 280 nm (D_{corr}) was measured as indicated in the main text, the results of which are given in Table S2.

Table S2 Reference decadic attenuance value determined for each lot of NISTmAb using UV-Vis spectrophotometry (path length, b = 0.05092 cm)

Lot	[a]Mean Decadic Attenuance (D_{corr})	Standard Deviation (Type A Uncertainty, SD_D)	Combined Standard Uncertainty (u_D)
**14HB-D-001**	0.72338	0.00066	0.00295
**14HB-D-002**	0.72331	0.00127	0.00319
**14HB-D-003**	0.71942	0.00099	0.00306
**PS 8670**	0.72721	0.00072	0.00297

[a]n = 10 for RM 8671 lots, n = 2 for PS 8670. Type A uncertainty is defined here as the standard deviation of replicate measurements with no propagation of error. Combined standard uncertainty is reported at a level of one standard deviation including full propagation of error.

A number of factors, in addition to the type-A measurement uncertainty (SD_D), contribute to the combined standard uncertainty (u_D). The Transfer Spectrophotometer (TS) used for these measurements is qualified against the NIST High Accuracy Spectrophotometer (HAS II) on a quarterly basis. Qualification of the TS at 280 nm is made through measurements on SRM 2031 absorbance filter, an SRM which was originally certified on the HAS II. Determination of the uncertainty for a TS measurement of SRM 2031 is described in the literature [2]. Contributions to the combined standard uncertainty based on this transfer control measurement include the

standard uncertainty of a single TS measurement of the SRM 2031 filter determined by simple replication (u_x), the combined standard uncertainty for the SRM 2031 reference standard as measured on the HAS II (u_{ref}), the bias of the transfer instrument (vs. HAS II) and the uncertainty associated with the bias determination (δ and u_δ, respectively). An additional term contributing to the uncertainty to photometric accuracy as a result of wavelength uncertainty (u_w) is also included. Traceability is ensured by including uncorrected bias in the measurement uncertainty rather than by bias correction [3, 4]. The bias itself must therefore be added to the uncertainty in the decadic attenuance [3, 4]. SRM 2031 utilized a coverage factor of 2; therefore, the combined standard uncertainty associated with the decadic attenuance is given by Equation S1.

$$u_D = \frac{|\delta| + 2\sqrt{u_{Dm}^2 + u_w^2 + u_x^2 + u_\delta^2 + u_{ref}^2}}{2} \qquad \text{(S1)}$$

The u_D was calculated according to Equation S1, followed by propagation of error through Equation 1 to achieve the combined standard uncertainty associated with the concentration measurement (u_c) as given in Table S3. The Type B contributions to the combined standard uncertainty budget are included in Table S4. The cuvette path length was measured previously by the NIST Dimensional Metrology Group using a Coordinate Measuring Machine fitted with a fiber probe as well as using interference fringes in the near-infrared, resulting in an additional Type B uncertainty (u_b). The uncertainty contribution associated with the theoretical extinction coefficient has not been fully evaluated. Instead the extinction coefficient has been utilized here as a constant, accepted industry norm value. For that reason, the mass concentration is reported as a reference value rather than a certified value.

Table S3 Reference mass concentration value determined for each lot of NISTmAb using UV-Vis spectrophotometry

Lot	[a]Mean Concentration (mg mL^{-1})	Standard Deviation (Type A Uncertainty, SD_c) (mg mL^{-1})	Combined Standard Uncertainty (u_c) (mg mL^{-1})
**14HB-D-001**	10.004	0.0091	0.0409
**14HB-D-002**	10.003	0.0176	0.0441
**14HB-D-003**	9.950	0.0137	0.0424
**PS 8670**	10.057	0.0099	0.0412

[a]*n = 10 for RM 8671 lots, n = 2 for PS 8670. Reference mas concentration is based on calculation via Equation 1, b = 0.05092 cm, and a theoretical extinction coefficient of 1.42* (mL mg^{-1} cm^{-1}). *Type A uncertainty is defined here as the standard deviation of replicate measurements with no propagation of error. Combined standard uncertainty is reported at a level of one standard deviation including full propagation of error.*

Table S4 Type B Uncertainty Components for Reference Mass Concentration Value Assignment

Uncertainty Components	Symbol	Value[(a)]	Uncertainty (Type)
Standard uncertainty associated with wavelength accuracy	u_w	0.001	Type B
Standard uncertainty of a single TS measurement on SRM 2031	u_x	0.000049	Type B
Combined standard uncertainty of SRM 2031	u_{ref}	0.0021	Type B
Standard uncertainty of the bias versus HAS II	u_δ	0.00026	Type B
Bias in transmittance density versus the HAS II	δ	0.0010	Type B
Combined standard uncertainty of the path length	u_b	0.000015[(b)]	Type B

[(a)] *Unless otherwise noted value is reported as arbitrary units (AU)*
[(b)]*Value is reported in centimeters.*

Physicochemical Reference and Informational Value Assignment and Homogeneity Samples. A total of nine samples were reserved for physicochemical homogeneity from each lot as depicted in Table S1 and Fig. S1. Samples were selected from racks across the fill sequence. The Combi dispenser (Thermo Scientific) described in [1] utilizes an 8 channel dispenser filling a given column simultaneously (e.g. column 1 rows A through H). Three samples from the same row in a given rack were selected in this matrix so that one sample from each row/rack#

combination could be dispensed to individual physicochemical assays. They were stored at –80 °C.

One vial from each lot/rack (1, 50, and 90) was thawed at room temperature for a total of three vials per lot. Each 800 µL vial was aliquoted into 5 x 150 µL fractions into a clean Thermo Matrix 1 mL tube. Each fraction was then frozen at –80 °C and stored until analysis by Capillary Zone Electrophoresis (CZE), Capillary Sodium Dodecylsulfate Electrophoresis (CE-SDS), Size Exclusion Chromatography (SEC), or LC-MS/MS peptide mapping. Method performance was evaluated on the day of analysis by injections of the method-specific instrument quality control standard (IQ) and PS 8670 as the system suitability standard that bracketed injections of RM 8671. The generic injection sequence was: Blank - IQ - PS 8670 - test samples (no more than 10) - IQ - PS 8670 – Blank, and was repeated as necessary to analyze all samples. Instrument qualification and system suitability controls were required to pass method performance criteria as outlined during method qualification [5, 6]. Replicates for each vial using CZE, CE-SDS, and SEC are as described in Table S5. The intended protocol for each lot was to perform triplicate repeatability on one vial (to obtain a measure of intra-vial repeatability) and individual measurements on two additional vials (to obtain a measure of inter-vial reproducibility) for physicochemical value assignment of each lot, as performed for CZE. CE-SDS followed this protocol for 14HB-D-001; however, fewer injections were performed on 14HB-D-002 and 14HB-D-003 to allow analysis within 24 hours such that the sample remained stable in the autosampler under reducing conditions. SEC analysis is an orthogonal measure of size heterogeneity; therefore replicates were performed to model CE-SDS with one sequence initiated immediately upon thaw and a second sequence initiated after storage at 4 °C for 24 hours post thaw in order to span a similar analysis timeframe.

Table S5 Replicates performed during physicochemical reference value assignment by method

	D-001			D-002			D-003		
**Vial from Rack #**	CZE	CE-SDS	SEC	CZE	CE-SDS	SEC	CZE	CE-SDS	SEC
**1**	3	3	6	1	1	2	1	1	2
**50**	1	1	2	3	1	2	1	1	2
**90**	1	1	2	1	1	2	3	1	2

To establish homogeneity and assign informational values, six vials (two obtained from each rack numbered 1, 50, and 90) from each of lot 14HB-D-001, 14HB-D-002 and 14HB-D-003 were analyzed by FI. Three vials from each lot 14HB-D-001, 14HB-D-002 and 14HB-D-003 were used for homogeneity assessment by DLS. One vial from each lot/rack (1, 50, and 90) was retained as a set of backup samples (not used for analysis), for a total of three vials per lot.

Statistical Treatment of Physicochemical Reference Values. The PS 8670 and RM 8671 materials are the same material (e.g. identical upstream and downstream production) other than processing steps performed during vialing. It is therefore reasonable to assume that individual methods will perform in a similar manner on both lots of material. In the first publication of this series, it was described how the method qualification exercises on PS 8670 were used to determine u^2_{other} representing intermediate precision components at a level of one standard deviation [1, 5, 6]. In the RM 8671 value assignment it was assumed that intermediate precision contributions (e.g. between column, inter-day, etc.) to total variance were identical.

$$u^2_{\text{other},70} = u^2_{\text{other},71} = u^2_{\text{other}} \tag{S2}$$

Each new lot of RM 8671 may also have contributions from the filling process (u_{fill}^2) and the repeatability of the analytical method (u_r^2). The inter-vial filling component must be included in RM 8671 measurements because samples were analyzed from three racks across the fill sequence. The combined standard uncertainty for RM 8671 measurements is therefore given by Equation S3.

$$u_{c,71}^2 = u_{\text{fill},71}^2 + u_{r,71}^2 + u_{\text{other}}^2 \qquad \text{(S3)}$$

The within day contributions ($u_{iv}^2 = u_{\text{fill}}^2 + u_r^2$) to the variance cannot be independently measured as both factors are contributing to the within day measurement. Therefore, a one-way nested ANOVA is used to calculate contributions to the within day precision and the total within day inter-vial variation. Substituting ($u_{iv}^2 = u_{fill}^2 + u_r^2$) we get:

$$\therefore u_{c,71}^2 = u_{iv,71}^2 + u_{\text{other}}^2 \qquad \text{(S4)}$$

Where u^2_{iv} can be treated as a Type A uncertainty for RM 8671, as it was measured.

u_{other}^2 is a Type B uncertainty, as it is a reference value from previous qualification [5, 6]. SEC, CE-SDS, and CZE uncertainty analyses were performed using this method to provide a combined standard uncertainty for RM 8671 (u_c) that incorporated PS method qualification experience and represents the intermediate precision at a level of one standard deviation. Reference values and associated combined standard uncertainty for each physicochemical assay are given in Table S6 through Table S8.

Table S6 CZE reference charge heterogeneity values for PS 8670 and RM 8671

	[a]PS 8670	[b]14HB-D-001	[b]14HB-D-002	[b]14HB-D-003
**Charge Purity (%)**	74.69 ± 0.34	73.81 ± 0.17	73.82 ± 0.17	73.75 ± 0.12
**Acidic Variants Relative Abundance (%)**	16.76 ± 0.40	16.52 ± 0.35	16.55 ± 0.39	16.55 ± 0.35
**Basic Variants Relative Abundance (%)**	8.54 ± 0.28	9.67 ± 0.22	9.62 ± 0.22	9.69 ± 0.21

[a] Stated uncertainty for PS 8670 represents the intermediate precision reported as a combined standard uncertainty, at a level of one standard deviation, based on ANOVA analysis for PS 8670 during qualification runs [5].

[b] Stated uncertainty for RM 8671 (n = 3 vials) represents the intermediate precision reported as a combined standard uncertainty, at a level of one standard deviation, based on ANOVA analysis.

Table S7 SEC reference size heterogeneity values for PS 8670 and RM 8671

	[a]PS 8670	[b]14 HB-D-001	[b]14 HB-D-002	[b]14 HB-D-003
**Monomeric Purity (%)**	98.78 ± 0.12	96.74 ± 0.14	96.63 ± 0.15	96.77 ± 0.14
**High Molecular Weight RA (%)**	1.02 ± 0.12	3.06 ± 0.13	3.17 ± 0.15	3.04 ± 0.14
**Low Molecular Weight RA (%)**	0.20 ± 0.008	0.20 ± 0.009	0.20 ± 0.008	0.20 ± 0.007

[a]Stated uncertainty for PS 8670 represents the intermediate precision reported as a combined standard uncertainty, at a level of one standard deviation, based on ANOVA analysis for PS 8670 during qualification runs [6].

[b] Stated uncertainty for RM 8671 (n = 3 vials) represents the intermediate precision reported as a combined standard uncertainty, at a level of one standard deviation, based on ANOVA analysis.

Table S8 CE-SDS reference size heterogeneity values for PS 8670 and RM 8671

	[a]PS 8670	[b]14HB-D-001	[b]14HB-D-002	[b]14HB-D-003
**Monomeric Purity (%) (nrCE-SDS)**	98.79 ± 0.38	98.49 ± 0.30	98.47 ± 0.79	98.14± 0.31
**Thioether RA (%) (rCE-SDS)**	0.31± 0.02	0.31± 0.01	0.30 ± 0.02	0.30 ± 0.005
**Glycan Occupancy (%) (rCE-SDS)**	99.40 ± 0.01	99.38 ± 0.01	99.39 ± 0.003	99.40 ± 0.01

[a]Stated uncertainty for PS 8670 represents the intermediate precision reported as a combined standard uncertainty, at a level of one standard deviation, based on ANOVA analysis for PS 8670 during qualification runs [6].

[b] Stated uncertainty for RM 8671 (n = 3 vials) represents the intermediate precision reported as a combined standard uncertainty, at a level of one standard deviation, based on ANOVA analysis.

In addition to the calculated physicochemical reference values listed in Table S8, it was determined that method performance (migration times and relative abundance) of individual, resolved components (monomer, light chain, heavy chain, etc.) may be useful to the stakeholder in comparing performance of orthogonal assays likely to be developed with the NISTmAb. Therefore additional Tables S9, S10, S11, and S12 are included in the ESM for PS 8670 (from the qualification exercise) and RM 8671 lots 14HB-D-001, 14HB-D-002, and 14HB-D-003 respectively.

Table S9 PS 8670 CE-SDS method performance [6].

Non-Reduced Samples

	Migration Time (min)	Relative Abundance (%)
	**Mean**	**Mean**
**Monomer**	28.19 ± 0.28	98.79 ± 0.38

Reduced Samples

	Migration Time (min)	Relative Abundance (%)
	**Mean**	**Mean**
**Light Chain**	15.29 ± 0.18	32.02 ± 0.20
**Heavy Chain**	19.29 ± 0.23	67.26 ± 0.20
**LC + HC**	N/A	99.28 ± 0.02
**Aglycosylated HC**	18.80 ± 0.22	0.40 ± 0.01
**HC Glycan Occupancy**	N/A	99.40 ± 0.01
**Thioether**	22.08 ± 0.27	0.31 ± 0.02

Stated uncertainty for PS 8670 represents the intermediate precision reported as a combined standard uncertainty, at a level of one standard deviation, based on ANOVA analysis for PS 8670 during qualification runs [6].

Table S10 RM 8671 Lot 14HB-D-001 CE-SDS method performance

Non-Reduced Samples

	Migration Time (min)	Relative Abundance (%)
	**Mean**	**Mean**
**Monomer**	27.99 ± 0.30	98.39 ± 0.47

Reduced Samples

	Migration Time (min)	Relative Abundance (%)
	**Mean**	**Mean**
**Light Chain**	15.16 ± 0.18	32.09 ± 0.20
**Heavy Chain**	19.11 ± 0.23	67.17 ± 0.20
**LC + HC**	N/A	99.27 ± 0.02
**Aglycosylated HC**	18.65 ± 0.23	0.42 ± 0.01
**HC Glycan Occupancy**	N/A	99.38 ± 0.01
**Thioether**	21.90 ± 0.27	0.31 ± 0.01

Stated uncertainty for RM 8671 (n = 3 vials) represents the intermediate precision reported as a combined standard uncertainty, at a level of one standard deviation, based on ANOVA analysis.

Table S11 RM 8671 Lot 14HB-D-002 CE-SDS method performance

Non-Reduced Samples

	Migration Time (min)	Relative Abundance (%)
	**Mean**	**Mean**
**Monomer**	28.32 ± 0.30	98.47 ± 0.79

Reduced Samples

	Migration Time (min)	Relative Abundance (%)
	**Mean**	**Mean**
**Light Chain**	15.34 ± 0.19	32.11 ± 0.21
**Heavy Chain**	19.35 ± 0.23	67.18 ± 0.20
**LC + HC**	N/A	99.28 ± 0.02
**Aglycosylated HC**	18.88 ± 0.23	0.41 ± 0.002
**HC Glycan Occupancy**	N/A	99.39 ± 0.003
**Thioether**	22.17 ± 0.28	0.30 ± 0.02

Stated uncertainty for RM 8671 (n = 3 vials) represents the intermediate precision reported as a combined standard uncertainty, at a level of one standard deviation, based on ANOVA analysis.

Table S12 RM 8671 Lot 14HB-D-003 CE-SDS method performance

Non-Reduced Samples

	Migration Time (min)	Relative Abundance (%)
	**Mean**	**Mean**
**Monomer**	28.51 ± 0.29	98.14 ± 0.31

Reduced Samples

	Migration Time (min)	Relative Abundance (%)
	**Mean**	**Mean**
**Light Chain**	15.43 ± 0.18	32.20 ± 0.20
**Heavy Chain**	19.46 ± 0.23	67.09 ± 0.19
**LC + HC**	N/A	99.30 ± 0.01
**Aglycosylated HC**	18.99 ± 0.22	0.41 ± 0.01
**HC Glycan Occupancy**	N/A	99.40 ± 0.01
**Thioether**	22.30 ± 0.27	0.30 ± 0.005

Stated uncertainty for RM 8671 (n = 3 vials) represents the intermediate precision reported as a combined standard uncertainty, at a level of one standard deviation, based on ANOVA analysis.

Informational Value Tables

Table S13 Subvisible particle concentrations (ECD ≥ 2 µm) in the controls (DIUF water, buffer, 5 µm commercial count standard), PS 8670, and RM 8671 material are shown

**Samples**	**[a]Particle Concentration (ECD ≥ 2 µm) (mL^{-1}), *n* ≥ 3**	**Range of Concentration of Protein in Particles (ng/mL)**	**[b]Range of Protein Mass in Particles/Protein Mass in Solution (%)**
**DIUF water**	12 (5)	-	-
**Buffer**	42 (32)	-	-
**5 µm count standard**	2898 (32)	-	-
**PS 8670**	4271 (1413)	105 to 449	0.0013 to 0.0045
14HB-**D-001**	4877 (1917)	56 to 540	0.00056 to 0.0054
14HB-**D-002**	6068 (1696)	85 to 287	0.00085 to 0.0029
14HB-**D-003**	6413 (2676)	84 to 524	0.00084 to 0.0053

[a]Each lot was analyzed in ≥ 3 runs with the uncertainty expressed as (SD). PS 8670 was analyzed in 4 runs from 4 different vials; 14HB-D-001 was analyzed in 5 runs from 5 different vials; 14HB-D-002 and 14HB-D-003 were analyzed in 6 runs from 6 different vials each.

[b]Total protein concentrations from referenced UV absorbance values were used to approximate the ratio of the concentration of protein in the particles to the concentration of protein in solution. The total protein concentration of PS 8670 was assumed to equal that of 14HB-D-001 (10.004 mg/mL).

Table S14 The Z-average hydrodynamic diameter obtained for the three lots of the RM 8671 material

**Lot**	Hydrodynamic Diameter (nm)
**PS 8670**	10.17 (0.19)
**14HB-D-001**	9.92 (0.47)
**14HB-D-002**	9.96 (0.31)
**14HB-D-003**	9.83 (0.17)

Three vials from each lot were sampled in triplicate and the uncertainty is expressed as (SD).

Thaw/Freeze Stability Samples. Intra-lot homogeneity analysis was performed as described in the main text (see Physicochemical Methods); therefore vials reserved for T/F stability were obtained from Rack 1 of 14HB-D-001 after homogeneity samples were removed. All vials were thawed at room temperature, and thaw/freeze cycles were performed, with the samples evenly split between –20 °C and –80 °C as described in Table S15. These temperatures were selected for T/F stability to represent the range of temperatures commonly employed for long term storage of frozen solutions.

Table S15 Thaw/Freeze Samples

**Step**	***Number of –80 °C Samples***	***Number of –20 °C Samples***	***5 vials labeled as:***	***From 1 vial make 150 µL x 5 aliquots and label as:***
**Thaw 0**	30	30	1FZ	1FZ_150 µL
**Freeze 1**	30	30		
**Thaw 1**	24	24	2FZ	2FZ_150 µL
**Freeze 2**	24	24		
**Thaw 2**	18	18	3FZ	3FZ_150 µL
**Freeze 3**	18	18		
**Thaw 3**	12	12	4FZ	4FZ_150_ µL
**Freeze 4**	12	12		
**Thaw 4**	6	6	5FZ	5FZ_150 µL
**Freeze 5**	6	6		

After each Thaw cycle one vial was inverted five times to mix, divided into 150 µL aliquots, labeled as “XFZ_150 µL” and then frozen at –80 °C for use in CZE, CE-SDS, SEC, and peptide mapping. An additional five vials from each T/F cycle were labeled as “XFZ,” frozen at –80 °C and retained for flow imaging and DLS. All samples were then refrozen. After

each refreeze, the labeled vials remained in the frozen state and unlabeled vials were thawed in the next round. Therefore the label indicates the total number of thaw/freeze cycles the sample underwent beyond the necessary initial thaw. The same sequence was performed simultaneously on 15 PS 8670 vials at each temperature (with the exception of three vials removed at each round) for a direct comparison during flow imaging analysis. All vials were stored at −80 °C after completion of the thaw/freeze cycles. The reference value (UV-Vis, CZE, SEC, and nrCE-SDS) or informational value (FI and DLS) for lot 14HB-D-001 was used as the zero T/F control.

Accelerated Stability Samples. Intra-lot homogeneity analysis was performed as described in the main text (see Physicochemical Methods); therefore vials reserved for accelerated stability were obtained from Rack 1 and Rack 2 of 14HB-D-001 after homogeneity samples were removed. At the appropriate sample start time, the indicated number of vials (Table S16) was thawed at room temperature for thirty (30) min and inverted five times to mix. They were then labeled “X Temperature_XXDays” to indicate the designated temperature condition and total exposure time and placed directly at the indicated temperature. The samples remained at the indicated accelerated stability temperature until Day 28.

Table S16 Accelerated Stability Samples

Sample start time	4 °C (number of samples)	Room temperature (number of samples)	40 °C (number of samples)	Total sample exposure time
**0 hours**	6	6	10	28 days
**Day 14**	6	6	6	14 days
**Day 21**	6	6	6	7 days
**Day 25**	6	6	6	4 days
**Day 27**	6	6	6	25 hours
**Day 28**	6			0 hours

Note that the 0 hours sample was prepared on Day 28. Multiple temperature conditions are not necessary for this sample as it is meant to be a zero-point control for all samples treated in the same manner, but not incubated. On Day 28, one of the vials from each condition was inverted five times to mix, divided into 150 µL aliquots, labeled as "X Temperature_XXDays" and then frozen at –80 °C. The remaining vials from each condition were frozen at –80 °C.

Sample Preparation Instrumentation. The 40 °C accelerated stability samples were heated in a VWR Forced Air Incubator. The 4 °C samples were incubated in a LabRepCo Futura refrigerator. The –20 °C samples were frozen in a Fisherbrand Manual Defrost Freezer. The samples were stored at –80 °C in a Forma –80 °C freezer.

Table S17 Method-Based Alternate Storage Conditions for RM 8671 Reference Value Determinations[a]

**Method**	**Attribute**	**Recommended Storage**	**Max T/F[b] (cycles)**		**Max Storage 4 °C (days)**	**Control Range**
			**–80 °C**	**–20 °C**		
**UV[c]**	Concentration	–80 °C	5	5	28	± 2u_c
**SEC[d]**	Monomeric Purity	–80 °C	5	5	7	± 3u_c
**nrCE-SDS[e]**	Monomeric Purity	–80 °C	5	5	28	± 3u_c
**rCE-SDS[f]**	Glycan Occupancy, Thioether Content	–80 °C	5	5	28	± 3u_c
**CZE[g]**	Charge Purity	–80 °C	5	5	28	± 3u_c

[a]Measured values are expected to be within the indicated control range, where u_c is the combined standard uncertainty, based on the alternate storage conditions listed for each individual method.
[b]Thaw/freeze cycles (T/F)
[c]Ultraviolet visible spectrophotometry (UV)
[d]Size exclusion chromatography (SEC)
[e]Non-reduced capillary sodium dodecyl sulfate electrophoresis (nrCE-SDS)
[f]Reduced capillary sodium dodecyl sulfate electrophoresis (rCE-SDS)
[g]Capillary zone electrophoresis (CZE)

Table S18 Method-Based Alternate Storage Conditions for RM 8671 Information Value Determinations and Peptide Mapping

**Method**	**Attribute**	**Recommended Storage**	**Max T/F[a] (cycles)**		**Max Storage 4 °C (days)**
			**–80 °C**	**–20 °C**	
**FI[b]**	Subvisible Particle Content	–80 °C	0	0	28
**DLS[c]**	Hydrodynamic Diameter	–80 °C	5	5	28
**Peptide Map**	Identity	–80 °C	ND[d]	ND	ND

[a]Thaw/freeze cycles (T/F)
[b]Flow imaging analysis (FI)
[c]Dynamic light scattering (DLS)
[d]Not determined

References

1. Schiel JE, Turner A. The NISTmAb Reference Material 8671 lifecycle management and quality plan. Anal Bioanal Chem. 2018; https://10.1007/s00216-017-0844-2.
2. Travis JC, Smith MV, Choquette SJ, Liu H-K. NIST Technical Note 1715: Certified Transmittance Density Uncertainties for Standard Reference Materials using a Transfer Spectrophotometer. 2011.
3. Magnusson B, Ellison SL. Treatment of uncorrected measurement bias in uncertainty estimation for chemical measurements. Anal Bioanal Chem. 2008;390:201-13. doi:10.1007/s00216-007-1693-1.
4. Synek V. Attempts to include uncorrected bias in the measurement uncertainty. Talanta. 2005;65(4):829-37. doi:10.1016/j.talanta.2004.07.038.
5. Turner A, Schiel JE. Qualification of NISTmAb charge heterogeneity control assays. Anal Bioanal Chem. 2018; https://10.1007/s00216-017-0816-6.
6. Turner A, Yandrofski K, Telikepalli S, King J, Keckert A, Filliben J et al. Development of orthogonal NISTmAb size heterogeneity control methods. Anal Bioanal Chem. 2018; https://10.1007/s00216-017-0819-3.
